# Supplementary material for: Drone images afford more detections of marine wildlife than real-time observers during simultaneous large-scale surveys
Source: PeerJ. 2023 Nov 3;11:e16186. doi: 10.7717/peerj.16186 (PMC10629383; doi:10.7717/peerj.16186)
Supplement: Supplemental Information 1 [file peerj-11-16186-s001.docx]

**Supplemental Article S1**

# Drone methods based on the Barnas et al. (2020) reporting protocol

# **1. Project Overview**

The objective of this research was to determine the efficacy of using drones for marine fauna surveys, and during this study we focused on dugongs (*Dugong dugon*). Our main objective was to test the assumption that drone imagery could provide comparable detection rates of dugongs to human observers on-board aircraft. We flew a light aircraft (Partenavia 68B) with a survey leader and four observers, and a *ScanEagle* drone over the same survey flight path at the same time, and were therefore able to compare detection rates for the same environmental conditions.

The surveys involved flying our drone beyond visual line of sight (BVLOS) along a series of parallel lines and continuously capturing still images of along those lines. The still images needed to cover a 400 m swath to replicate the 2 x 200 m strips being surveyed by the observers, and the total area surveyed was around 600 km^2^.

# **2. Drone System and Operation Details**

## *2.1 Platform specifications*

We used the *ScanEagle* UAV, which was owned and operated by Insitu Pacific Ltd (IPL). This is a fixed-wing UAV and provides relatively large range (up to 100 km, extended using relay stations) and long endurance (24+ hrs) capabilities. Table 1 provides the details of the *ScanEagle* (as at the time we used this system in 2012; see <https://insitupacific.com.au/> for current systems and specifications). We had three *ScanEagle* systems for redundancy in the case of system failures and to allow us to launch a second system immediately upon retrieving the first, when we wanted to conduct multiple flights in one day.

Table S1. Specifications of the ScanEagle UAV used in our study.

| Dimensions | Wing Span | 10.2 ft | 3.11 m |
| --- | --- | --- | --- |
|  | Length | 4.5 ft | 1.37 m |
| Weights | Empty Structure Weight | 28.8 lb | 13.1 kg |
|  | Max Take-off Weight | 44.0 lb | 20.0 kg |
| Performance | Max Horizontal Speed | 80 knots | 41 m/s |
|  | Cruise Speed | 48 knots | 25 m/s |
|  | Ceiling | 19500 ft | 5944 m |
|  | Endurance | 24+ hours | |
| System features | Propulsion | 1.9 hp (1.4 kw), 2-stroke engine | |
|  | Fuel | Gasoline (100 octane unleaded non-oxygenated gas) | |
|  | Navigation | GPS / Inertial | |
|  | Launch | Pneumatic catapult (“Superwedge UAV Launcher”) | |
|  | Recovery | SkyHook wingtip capture (“Skyhook”) | |

## *2.2 Take off and retrieval*

The *ScanEagle* was deployed using a pneumatic catapult “Superwedge” launcher and recovered using the “Skyhook” retrieval system, both of which were set up at Monkey Mia airport (Shark Bay) next to the ground control station (GCS). This equipment required a flat area large enough for the footprint of the GCS, the Superwedge and the Skyhook, and clear from obstruction in the direction of launching and retrieving the *ScanEagle*. The retrieval involved the *ScanEagle* flying into the vertical line on the Skyhook which then was caught by a clip on the end of the wing (Figure 1).

**
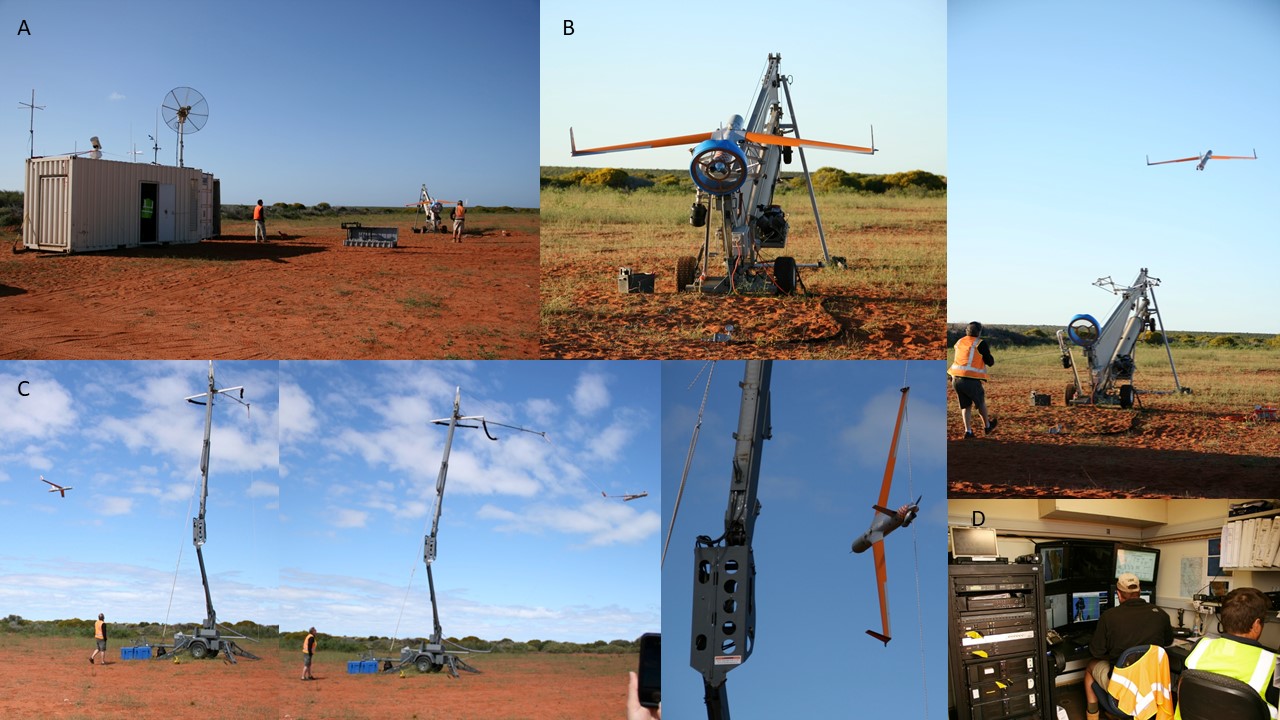
**

Figure S1. A) Ground control station at Monkey Mia airport, including B) the “Superwedge” launcher, C) Skyhook retrieval system, and D) the controls inside the GCS.

## *2.3 Flight planning and method of operation*

The *ScanEagle* was operated by six personnel from IPL. They have their own flight planning and operational software which is controlled under International Traffic in Arms Regulations. We provided IPL with the waypoints for our survey flight plan, which was designed using ArcGIS® software by Esri (Figure 2). During the survey flights, the *ScanEagle* flew autonomously and was tracked in real time via the operational software.


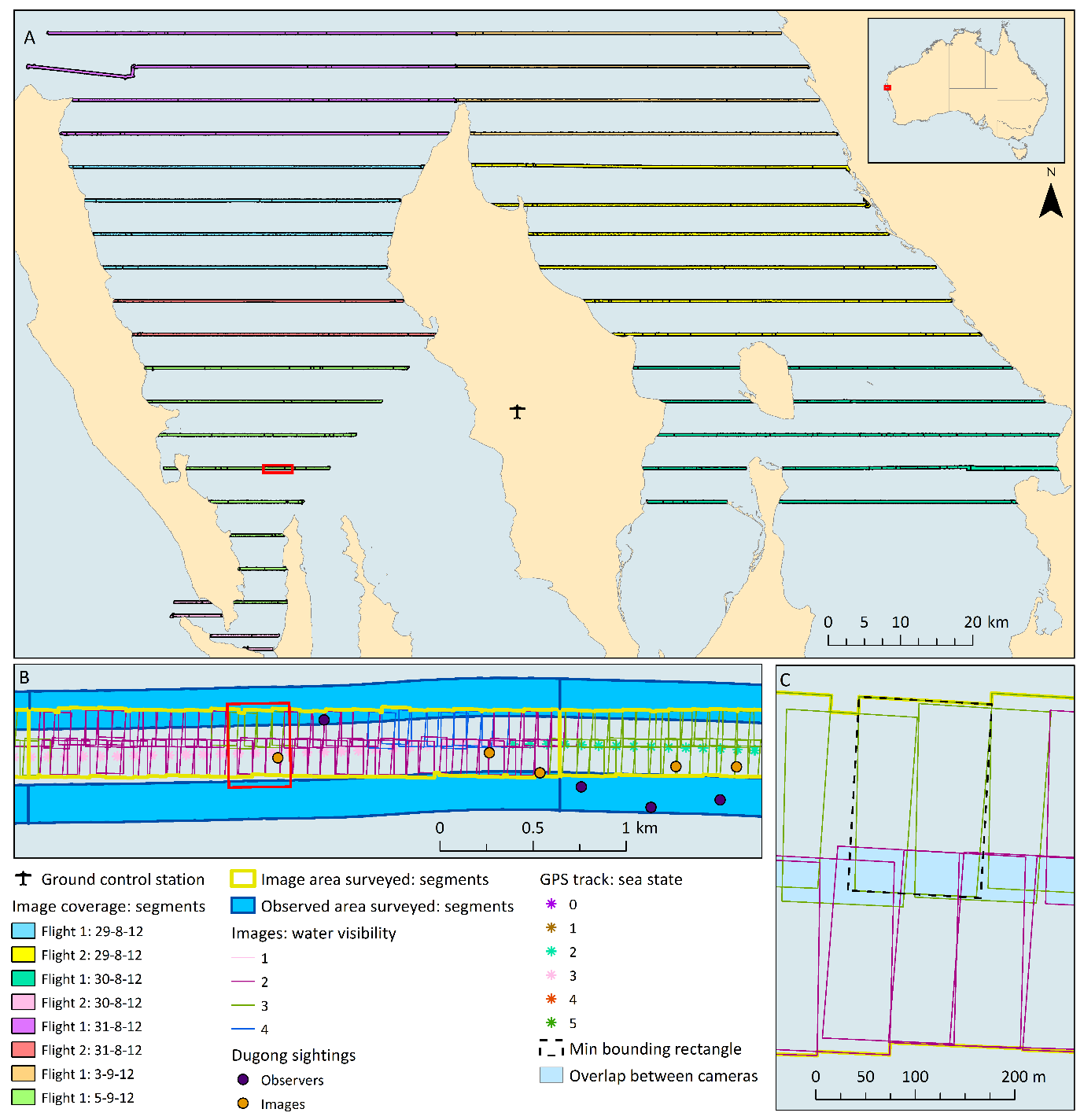


Figure 2. Red extent indicators show the extent of the map of subsequent resolutions. (A) Coverage of images along each transect (split into segments) for each flight. Note that the area covered by flight 1, 29^th^ was also covered within flight 2, 31^st^, and the area covered by flight 1, 5^th^ was also covered within flight 2, 30^th^. (B) Example of the coverage from each platform along a portion of a transect, showing the splitting of the transect into segments of the same length for each platform according to water visibility scores from the images. The GPS track is shown with each point given a sea state score, which were averaged within each segment. Also showing dugong sightings mapped accurately within the images and coarsely from the manned sightings. (C) Closer view of images mapped and the calculated of total area surveyed, side-lap between the cameras, and the on-ground width of images according to a minimum bounding rectangle.

**3. Payload, Sensor, and Data Collection**

*3.2 Payload and sensor description*

We had a target ground sample distance (GSD – distance between pixel centres measured on the ground) of at least 3 cm to allow detection of dugongs. We used two digital SLR cameras (24 megapixel (6016 x 4000) Nikon® D3200), each fitted with a standard 50 mm lens and a polarising filter set so that the direction of the polarisation was kept constant. Each camera was set at ~ 11.5° from vertical (nadir) in opposite directions, and we flew at 1400 ft (427 m) to achieve a combined swath of ~400 m (though we dropped the flight altitude to 1300 ft (396 m) for some flights to increase the resolution slightly).

We had a fixed video camera in the nose of the *ScanEagle* from which imagery was viewed in real-time from the GCS, providing improved situational awareness. The SLR cameras were mounted within the airframe using a number of shock-absorbing mounts to reduce vibrations.

*3.1 Data collection*

A summary of the data collection is provided in Table 1.

We collected still images (RAW format) which were stored on the cameras’ memory cards and downloaded post flight. It was important for our research purposes to be able to accurately georeference every image captured during our surveys, so IPL customized their system such that the rotations of the *ScanEagle* at the time of image capture was written to the EXIF data of each image (which was also tagged with time to nearest millisecond and a GPS location).

Image capture was controlled (including start, stop and capture rate) via the GCS and the capture rate could be scheduled to achieve a prescribed 40% image forward-lap (overlap between successive images) ensuring complete coverage along the transect lines flown. All images were stored on the camera’s memory card and downloaded post flight.

The camera settings were altered daily according to weather conditions and the output of the previous flights.

**4. Field Operation Details**

We conducted nine *ScanEagle* flights during eight days between 29 August and 5 September 2012. Details of the flights are provided in Table 1. Wind conditions varied throughout the flights, with Beaufort sea state ranging from 1 to 5. The target ground speed was 60 knots.

During one flight the *ScanEagle* lost contact with the GCS while surveying the second most north-western transect. The *ScanEagle* is preprogramed to return to the GCS when this situation arises, but just after it started heading to base we regained communications and sent the *ScanEagle* back to its original flight path (i.e. back on transect), and continued the remainder of the flight without incident (see Figure 2). As the *ScanEagle* had only gone slightly off track and continued capturing images, we included this section in the analysis.

Limits imposed by our *ScanEagle* system set-up included a maximum flying time of approximately 5 hrs according to the environmental conditions and fuel load capacity (which was limited by the weight of the camera payloads). Each SLR camera held a 128 GB memory card which allowed us to conduct at least five hours of surveying.

**Table 2. Summary of drone flights.**

| Flight | UAV Altitude (ft/m) | Ground sample distance (cm) | Total transects | Total length of transects (km) | UAV coverage details | | | Area covered (km2) | Images on "effort" | Camera settings |
| --- | --- | --- | --- | --- | --- | --- | --- | --- | --- | --- |
|  |  |  |  |  | Mean (SD) percentage forward-lap of images | Mean width of images (m) | Percentage of area side-lap between cameras* |  |  |  |
| 29F1 | 1400 / 427 | 3.5 | 4 | 157.54 | 39.5 (9.3) | 210.2 (1.9) | 13.8 | 64.4 | 4209 | Aperture priority F-stop 1.8, min shutter speed 1/2000s, ISO 100 auto (max 800) |
| 29F2 | 1400 / 427 | 3.5 | 6 | 281.91 | 40.7 (8.1) | 208.7 (1.5) | 13.4 | 114.8 | 7572 | Shutter speed priority set at 1/1000s, Aperture free to change, ISO auto |
| 30F1 | 1300 / 396 | 3.2 | 6 | 241.87 | 39 (11.2) | 199.7 (24.2) | 13.0 | 90.5 | 6815 | Aperture priority set at 2.5 F-stop, min shutter speed 1/500 and ISO auto |
| 30F2 | 1300 / 396 | 3.2 | 12 | 165.39 | 41.9 (9) | 193.4 (5.7) | 12.0 | 65.5 | 4750 | Shutter speed priority set at 1/1000s, Aperture free to change, ISO 200 (auto) |
| 31F1 | 1400 / 427 | 3.5 | 4 | 205.95 | 41 (8.1) | 206.2 (6.6) | 13.4 | 83.3 | 5509 | Shutter speed priority set at 1/1000s, Aperture free to change, ISO 200 (auto) |
| 31F2 | 1300 / 396 | 3.2 | 6 | 231.94 | 41 (9.1) | 193.9 (3.5) | 13.1 | 94.9 | 6697 | Aperture priority 1.8 f-stop, ISO 100 default & auto control, min shutter speed 1/1000 but auto |
| 3F1 | 1300 / 396 | 3.2 | 4 | 176.74 | 41.6 (9.3) | 194.4 (1.6) | 12.2 | 73.8 | 5089 | Aperture priority F-stop 1.8, min shutter speed 1/2000s, ISO 100 auto (max 800) |
| 5F1 | 1300 / 396 | 3.2 | 8 | 140.54 | 41.5 (8.8) | 193.9 (1.9) | 12.3 | 57.2 | 4053 | Aperture priority F-stop 1.8, min shutter speed 1/2000s, ISO 100 auto (max 800) |
| Total |  |  |  | 1601.87 |  |  |  | 645.3 | 44694 |  |

* This percentage overlap is based on the total area of coverage from each camera and the total area of overlap between the two.

**5. Data Post-Processing**

All survey images (in RAW format) were reviewed manually post flight by three marine mammal experts (two of whom were experienced aerial survey observers) who identified all dugongs (and cetaceans) in the images. We used the Nikon^®^ ViewNX 2™ software to review the images as this software is able to read the data of a RAW image and displayed the best ‘true-color’ version of the images.

We used custom software called VADAR (Visual Acoustic Detection and Ranging; Eric Kniest, University of Newcastle) to map the outline of all images using GPS data and drone rotation data (pitch, tilt and roll) written to each image at the time of capture. We also used VADAR to plot the GPS location for each individual dugong within the images. This process aided in validating the precision of the drone flight path, the realized coverage from the images, the overlap between images, and the double counts of dugongs. The latter was confirmed according to the GPS positions VADAR provided for each individual whale in the images and by plotting all sightings together.

**6. Permits, Regulations, Training, and Logistics**

IPL were responsible for obtaining Australian Civil Aviation Safety Authority (CASA) approval to operate the *ScanEagle* BVLOS, and their personnel held all the appropriate operational certification. CASA required that we operate under a valid NOTAM (Notice to Airmen). Additionally operations were to remain separated from any general traffic that overflies or enters the operating area by at least the safe separation distances of a minimum of 152 m vertically and 1500 m horizontally. We were required to have a certified aeronautical radio operator as part of the IPL team when we were operating BVLOS.

We were required to have an ethics permit to conduct the surveys over dugongs and cetaceans, along with a marine park permit to conduct research with Shark Bay Marine Park.

**References**

Barnas, A. F., D. Chabot, A. J. Hodgson, D. W. Johnston, D. M. Bird and S. N. Ellis-Felege (2020). "A standardized protocol for reporting methods when using drones for wildlife research." Journal of Unmanned Vehicle Systems **8**(2): 89-98. DOI: 10.1139/juvs-2019-0011.
