# Supplementary material for: Drone images afford more detections of marine wildlife than real-time observers during simultaneous large-scale surveys
Source: PeerJ. 2023 Nov 3;11:e16186. doi: 10.7717/peerj.16186 (PMC10629383; doi:10.7717/peerj.16186)
Supplement: Supplemental Information 2 [file peerj-11-16186-s002.docx]

**Supplemental Article S2**

# Perception Bias/Detectability Sample Size Calculations

#### Estimating detection probability

Double-observer configurations are used to estimate ‘perception bias’, or its opposite, ‘detectability’, which is an estimate of the probability an observer will detect an object/animal given it is present to be detected; the estimate of detection probability is then used to correct the detected counts to an estimate of the total number of objects/animals present (with or without any other bias corrections). This simulation exercise aims to explore the impact of number of detections, and rates of recapture, for a generic double-observer configuration. In particular, this simulation exercise seeks to find an ‘optimal’ or at least a ‘minimum’ number of detections that should be sought in order to estimate perception bias/detectability using double-observer data with a high level of precision and accuracy. In the context of a double-observer configuration to review aerial images that may contain animals (i.e., where imagery has been collected as part of an aerial imagery survey), this analysis does not consider the total number of images taken. Like line transect distance sampling, it’s the number of detections that helps with the quality of fit of a detection function; the total amount of survey effort will (hopefully!) have been designed to ensure an adequate or minimum number of detections were made.

In an initial modelling effort, double-observer configurations were simulated–the first, a true double-observer scenario, with two observers, and another with three observers. Assumed detection probabilities for each observer was set, ranging from 0.4 to 1.0, going up in increments of 0.1. Total number of detections for each experiment ranged from 5 up to 500, incrementing between 5 and 100 (increment lengths increasing as total number of detections increased). Each observer could have the same or different detection probability to the others in the scenario. In total there were 392 different combinations of detection probabilities and total number of detections for the two observers, and 2744 different combinations for the three-observer scenario.

Detection probability was estimated with a Huggins closed-capture model (Huggins 1989, 1991) using using MARK [Version 9.0; White and Burnham (1999)] via an RMark (Laake 2013) interface within R version 4.0.2 (R Core Team 2020). The first observer is considered a ‘capture’, and the second (and third, if applicable) observer is considered as a ‘recapture’ (at time step 1 or 2, as appropriate). The ‘closed-capture’ description refers to the assumption that no study subjects entered or exited the survey/sample area in the time in between when the individual observers do their counts (Otis et al. 1978). (This is a trivial point in image surveys, because an object/animal will not appear/disappear from an image in between observers having an opportunity to view that image; movement, however, can occur during surveys in the field.) Using matched detections from observers during a double-observer experiment, a capture history is created for each detection *k*, which would be (*x*^k^_11_) if both observers saw the *k*th object/animal, (*x*^k^_10_) if only observer 1 detected the object, and (*x*^k^_01_) if only observer 2 detected the object. (This notation is easily extened to a three-observer scenario.) Typically, the closed-capture model was fitted under the assumption there was no error in the matching process (mentioned here for completion).

The Huggins closed-capture model allows for the inclusion of covariates to help describe heterogeneity in detection probabilities. The impact of including simulated covariates on CVs can be considered in future if required, but these have not been specifically accounted for here.

Huggins closed-capture model provides an estimate of detection probability for a single observer, i.e., *p*_1_ = *p*_2_ = probability of detection for a single observer, under the assumption of equal detection probabilities. It can also provide an estimate of detection probability for unique observers. It is also possible to consider an estimate of combined probability of detection (i.e., the probability that at least one observer detects the object/animal), but that specific parameter is not estimated here. This can be further explored in the future if needed.

The R code to simulate ‘estimation’ of the capture probabilities (i.e, detection probabilities) for the *TWO* observers is given below. Nominated detection probabilities did not dip below 0.4 as I thought that was a reasonable minimum (i.e., something has gone a bit wrong of detection probability is getting below that). We can easily nominate lower detection probabilities in a future iteration.

huggins.sim.f<- function(i, p.e)
{

#grab a single row of the simulation set up data frame
 p.e.i<- p.e[ i,]

 #pull out the total detections, and nominated detection probabilities for observers 1 and 2
 total.detections<- p.e.i$total.detections
 detections.1<- p.e.i$p.1.e
 detections.2<- p.e.i$p.2.e

 #estimate the probabilities of the '10', '01' and '11' capture histories, according to the nominated detection probabilties above.
 p.ch.10<- detections.1*(1-detections.2)
 p.ch.01<- detections.2*(1-detections.1)
 p.ch.11<- detections.1*detections.2
 p.combo<- 1-((1-detections.1)*(1-detections.2)) #estimate the combined probability of the '10', '01' and '11' capture history, to apply Bayes' Theorem below.

 #estimate the relative probabilities of each capture history (due to the fact that '00' has been removed from the sample space as it's an unobservable state)
 c.p.ch.10<- p.ch.10/p.combo
 c.p.ch.01<- p.ch.01/p.combo
 c.p.ch.11<- p.ch.11/p.combo

 #create a capture histories in the format the RMark requires
 ch<- c( rep("01", round( c.p.ch.01*total.detections)), rep("10", round( c.p.ch.10*total.detections)), rep("11", round( c.p.ch.11*total.detections)))

 rmark.data.ready<- data.frame( ch= ch)

 #set up for Huggins closed-population mark-recapture analysis, where observers are specified with a unique capture probability (detection probability in this context)
 x.proc.obs<- process.data( rmark.data.ready, model="Huggins", groups= NULL)
 x.ddl.obs<- make.design.data( x.proc.obs)

 p.sharetime=list(formula=~time, share=TRUE)
 mod.1=mark(x.proc.obs, x.ddl.obs, model.parameters=list(p=p.sharetime), invisible=TRUE, output=FALSE, silent=TRUE)

 rr.reporting=get.real( mod.1,"p",se=TRUE,vcv=TRUE)


 #pull out the estimated parameters, standard errors and estimated CVs and summarise in a single table.
 ans<- data.frame( p.1.e= detections.1, p.2.e=detections.2, total.detections=total.detections, p.1.hat=rr.reporting$estimates$estimate[1], p.2.hat=rr.reporting$estimates$estimate[2], p.1.hat.se=rr.reporting$estimates$se[1], p.2.hat.se=rr.reporting$estimates$se[2], p.1.hat.cv = rr.reporting$estimates$se[1]/rr.reporting$estimates$estimate[1], p.2.hat.cv = rr.reporting$estimates$se[2]/rr.reporting$estimates$estimate[2])

 return( ans)

}


#set up nominated detection probabilities and total number of detections. Permutates so all levels are combined with all other levels of each variable.
p.e.2<- expand.grid(seq(0.4,1,0.1), seq(0.4,1,0.1), c(5,10,20,50,75,100,200,500))

colnames(p.e.2)<- c("p.1.e","p.2.e","total.detections")


#huggins.sample.size.sim.1<- do.call(rbind, pblapply( 1:nrow(p.e), huggins.sim.f, p.e = p.e.2))

cleanup(lx = NULL, ask = FALSE, prefix = "mark")

The R code to simulate ‘estimation’ of the capture probabilities (i.e, detection probabilities) for the *THREE* observers is given below.

huggins.sim.3obs.f<- function(i, p.e)
{

 #grab a single row of the simulation set up data frame
 p.e.i<- p.e[ i,]

 #pull out the total detections, and nominated detection probabilities for observers 1, 2 and 3
 total.detections<- p.e.i$total.detections
 detections.1<- p.e.i$p.1.e
 detections.2<- p.e.i$p.2.e
 detections.3<- p.e.i$p.3.e

 #estimate the probabilities of the '100', '101', '110', '111', '010', '011', '001' capture histories, according to the nominated detection probabilties above.
 p.ch.100<- detections.1*(1-detections.2)*(1-detections.3)
 p.ch.101<- detections.1*(1-detections.2)*detections.3
 p.ch.110<- detections.1*detections.2*(1-detections.3)
 p.ch.111<- detections.1*detections.2*detections.3
 p.ch.010<- (1-detections.1)*detections.2*(1-detections.3)
 p.ch.011<- (1-detections.1)*detections.2*detections.3
 p.ch.001<- (1-detections.1)*(1-detections.2)*detections.3

 p.combo<- 1-((1-detections.1)*(1-detections.2)*(1-detections.3)) #estimate the combined probability of the '100', '101', '110', '111', '010', '011', '001'.

 #estimate the relative probabilities of each capture history (due to the fact that '00' has been removed from the sample space as it's an unobservable state)
 c.p.ch.100<- p.ch.100/p.combo
 c.p.ch.101<- p.ch.101/p.combo
 c.p.ch.110<- p.ch.110/p.combo
 c.p.ch.111<- p.ch.111/p.combo
 c.p.ch.010<- p.ch.010/p.combo
 c.p.ch.011<- p.ch.011/p.combo
 c.p.ch.001<- p.ch.001/p.combo


 #create a capture histories in the format the RMark requires
 ch<- c( rep("100", round( c.p.ch.100*total.detections)), rep("101", round( c.p.ch.101*total.detections)), rep("110", round( c.p.ch.110*total.detections)), rep("111", round( c.p.ch.111*total.detections)), rep("010", round( c.p.ch.010*total.detections)), rep("011", round( c.p.ch.011*total.detections)), rep("001", round( c.p.ch.001*total.detections)))


 rmark.data.ready<- data.frame( ch= ch)

 #set up for Huggins closed-population mark-recapture analysis, where observers are specified with a unique capture probability (detection probability in this context)
 x.proc.obs<- process.data( rmark.data.ready, model="Huggins", groups= NULL)
 x.ddl.obs<- make.design.data( x.proc.obs)

 p.sharetime=list(formula=~time, share=TRUE)
 mod.1=mark(x.proc.obs, x.ddl.obs, model.parameters=list(p=p.sharetime), invisible=TRUE, output=FALSE, silent=TRUE)

 rr.reporting=get.real( mod.1,"p",se=TRUE,vcv=TRUE)


 #pull out the estimated parameters, standard errors and estimated CVs and summarise in a single table.
 ans<- data.frame( p.1.e= detections.1, p.2.e=detections.2, p.3.e=detections.3, total.detections=total.detections, p.1.hat=rr.reporting$estimates$estimate[1], p.2.hat=rr.reporting$estimates$estimate[2], p.3.hat=rr.reporting$estimates$estimate[3], p.1.hat.se=rr.reporting$estimates$se[1], p.2.hat.se=rr.reporting$estimates$se[2], p.3.hat.se=rr.reporting$estimates$se[3], p.1.hat.cv = rr.reporting$estimates$se[1]/rr.reporting$estimates$estimate[1], p.2.hat.cv = rr.reporting$estimates$se[2]/rr.reporting$estimates$estimate[2], p.3.hat.cv = rr.reporting$estimates$se[3]/rr.reporting$estimates$estimate[3])


 return( ans)

}


#set up nominated detection probabilities and total number of detections. Permutates so all levels are combined with all other levels of each variable.
p.e<- expand.grid(seq(0.4,1,0.1), seq(0.4,1,0.1), seq(0.4,1,0.1), c(5,10,20,50,75,100,200,500))

colnames(p.e)<- c("p.1.e","p.2.e","p.3.e", "total.detections")


#huggins.3obs.sample.size.sim.1<- do.call(rbind, pblapply( 1:nrow(p.e), huggins.sim.3obs.f, p.e = p.e))

cleanup(lx = NULL, ask = FALSE, prefix = "mark")


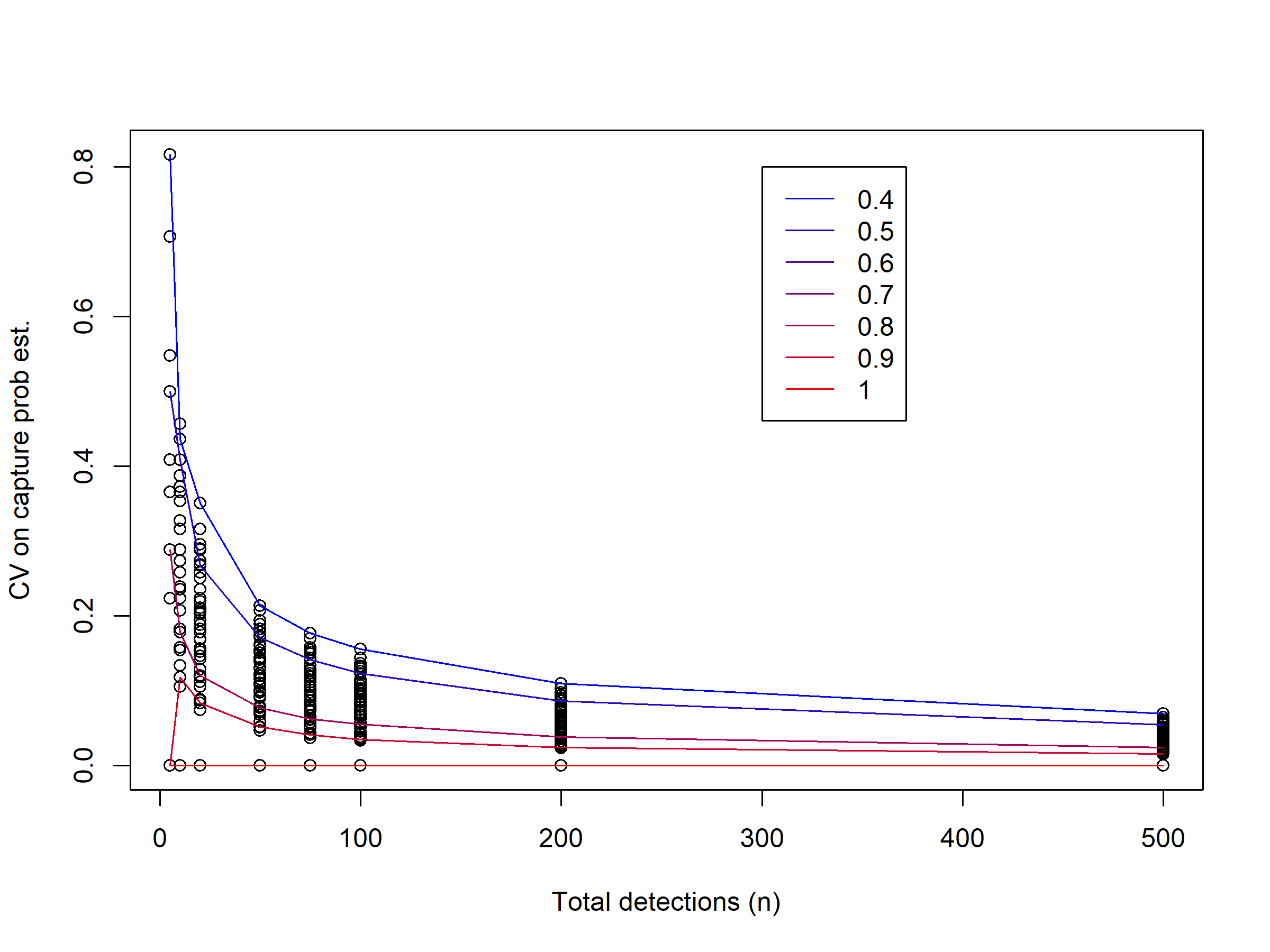


**Figure 1**: CV of the estimate of detection probability for a single observer for various nominated detection probabilities (given by lines of differing colours) and total number of detections, for a TWO-observer scenario. Lines represent combination of nominated detection probabilities are equal for the two observers (selected for convenience). Black circles represent other combinations of nominated detection probabilities.


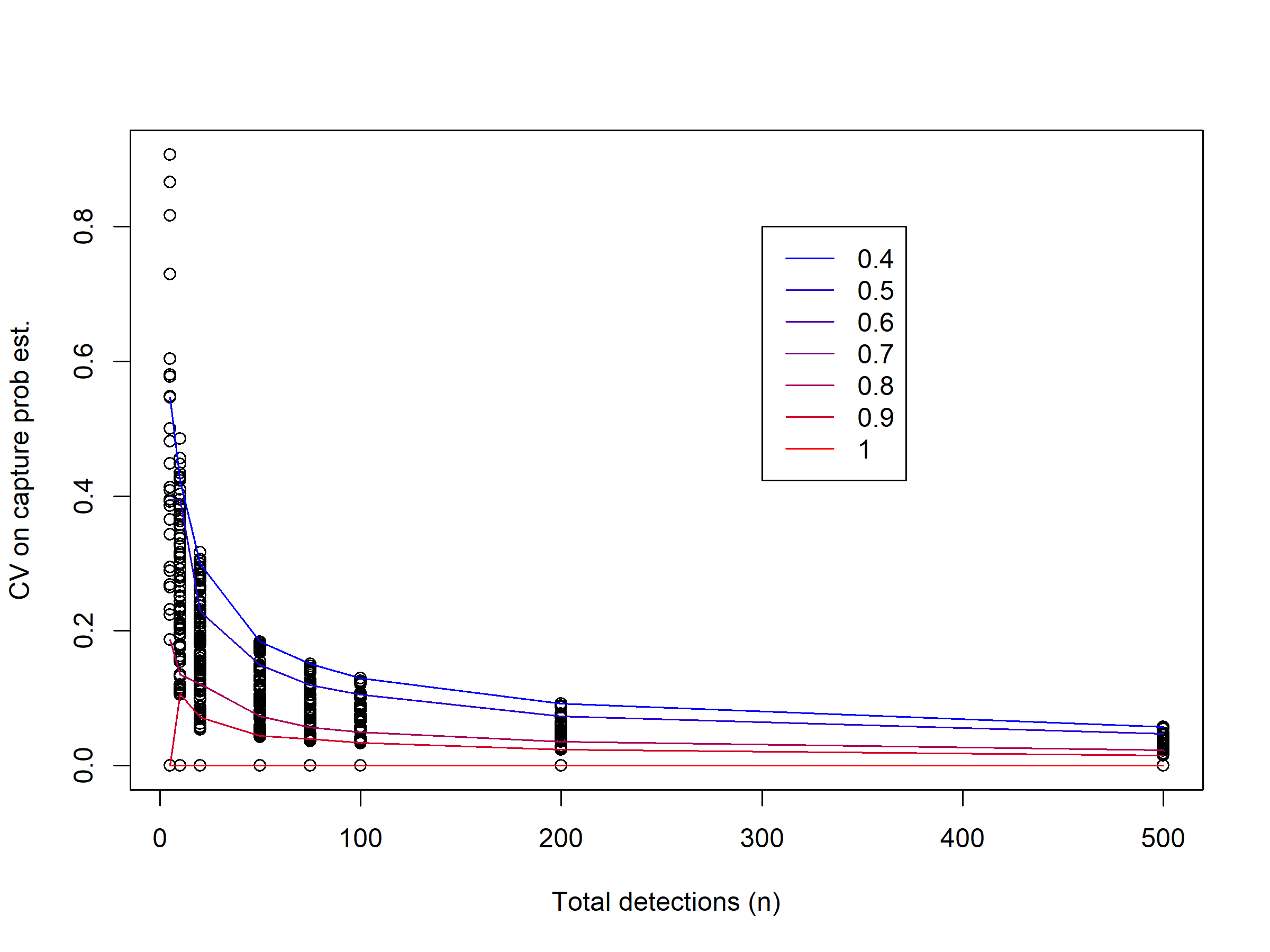


**Figure 2**: CV of the estimate of detection probability for a single observer for various nominated detection probabilities (given by lines of differing colours) and total number of detections, for a THREE-observer scenario. Lines represent combination of nominated detection probabilities are equal for the three observers (selected for convenience). Black circles represent other combinations of nominated detection probabilities.

It’s hard to nominate CV below which you’d like to aim; you just want it to be as low as possible under reasonable/realistic sampling effort. However, if you look at *Figure 1* (i.e., the two-observer scenario), you can see a point at which the CV does not really decrease that much as a function of increasing total number of detections, for each of the nominated detection probabilities. This rough ‘asymptote’ point is probably a good way to indicate the minimum number of detections you’d want to aim for to achieve the best (i.e., lowest) CV you can, but within reasonably achievable sample sizes. I would say, when not considering covariates (an analysis/simulation for another time?), roughly speaking, you should aiming for 80-100 unique detections combined between the two observers (based on the fact that CV doesn’t seem to decrease substantially after 100 detections, so insisting on more detections would run into the law of diminishing returns). Looks like it’s at as similar number of detections for the triple-observer scenario (see *Figure 2*), possibly slightly fewer at around 75? (In any case, the presence of a third observer in the observation process decreases the CVs by ~0.03-0.05, if roughly comparing *Figure 1* and *Figure 2* by eye.) Inclusion of covariates in the double-observer analysis may require more detections to achieve similar CVs.

#### References

Huggins, R. M. 1989. “On the Statistical Analysis of Capture Experiments.” Journal Article. *Biometrika* 76 (1): 133–40. <https://doi.org/10.1093/biomet/76.1.133>.

———. 1991. “Some Practical Aspects of a Conditional Likelihood Approach to Capture Experiments.” Journal Article. *Biometrics* 47 (2): 725–32. <https://doi.org/10.2307/2532158>.

Laake, J. L. 2013. “RMark: An r Interface for Analysis of Capture-Recapture Data with MARK.” {AFSC} Processed Rep. 2013-01. Seattle, WA: Alaska Fish. Sci. Cent., NOAA, Natl. Mar. Fish. Serv. <http://www.afsc.noaa.gov/Publications/ProcRpt/PR2013-01.pdf>.

Otis, David L., Kenneth P. Burnham, Gary C. White, and David R. Anderson. 1978. “Statistical Inference from Capture Data on Closed Animal Populations.” Journal Article. *Wildlife Monographs*, no. 62: 3–135. <http://pubs.er.usgs.gov/publication/70119899>.

R Core Team. 2020. *R: A Language and Environment for Statistical Computing*. Vienna, Austria: R Foundation for Statistical Computing. <https://www.R-project.org/>.

White, Gary C., and Kenneth P. Burnham. 1999. “Program MARK: Survival Estimation from Populations of Marked Animals.” Journal Article. *Bird Study* 46 (sup1): S120–39. <https://doi.org/10.1080/00063659909477239>.
