# Supplementary material for: Drone images afford more detections of marine wildlife than real-time observers during simultaneous large-scale surveys
Source: PeerJ. 2023 Nov 3;11:e16186. doi: 10.7717/peerj.16186 (PMC10629383; doi:10.7717/peerj.16186)
Supplement: Supplemental Information 3 [file peerj-11-16186-s003.docx]

**Supplemental Article S3**

Estimating perception probability for human observers on occupied and image-based aerial surveys

# Introduction

In visual count surveys, perception probability is the probability that an individual or group which is available to be seen by the observer(s) is actually detected by that observer; the obverse is known as perception bias, which is the probability an individual/group which is available to be seen is in fact missed (Marsh and Sinclair 1989). Double-observer methods allow for the estimation of perception probability via mark-recapture (Nichols et al. 2000). Multiple observers (at least two, which may be independent, but not necessarily so) are tasked at viewing the same or similar survey areas (whether in the field in real time or using survey imagery), and a capture history is built up regarding whether the observers made the same detections of individual/groups. Covariates which may potentially influence perception probability can also be included and tested in the mark-recapture model.

Double observer methods were used in Hodgson et al. to estimate perception probability of both human observers on occupied flights, and observers reviewing along-track images from the concurrent aerial image survey (taken from a drone).

## Methods and results

### Double-observer approach

Perception probability was estimated with a Huggins closed-capture model (Huggins 1989, 1991) using using MARK [Version 9.0; White and Burnham (1999)] via an RMark (Laake 2013) interface within R version 4.0.2 (R Core Team 2020). The first analyst is considered a ‘capture’, and the second (and third, in the case with the image review team) analyst were considered the ‘recapture’. The ‘closed-capture’ description refers to the assumption that no study subjects (dugong group detections, in this example) entered or exited the survey area or aerial imagery in the time in-between the individual observers do their counts (Otis et al. 1978)–entirely a given in the example of imagery data, but some movement/diving is theoretically possible in between the times of detection between the front and back observers of the occupied flights. A capture history was created for each detection *k*, which would be (*x*^k^_11_) if both analysts detected the *k*th group, (*x*^k^_10_) if only analyst 1 detected the group, etc (easily extended to an n-observer example). The closed-capture model was fitted under the assumption there was no error in the matching process.

The Huggins closed-capture model allows for the inclusion of covariates to help describe heterogeneity in detection probabilities, and these were included in the double-observer analyses; specific details below.

### Aerial imagery observers

Three observers (referred to in the manuscript as reviewers to distinguish them from the observer onboard the aircraft) viewed the 3490 images from the drone surveys, within which there were 96 detections of dugong groups. Several potentially influential covariates were recorded for aerial images containing dugong group detections, including water visibility (turbidity), glare, group size and the observer ID in order to test their effect on detection probability.

Water visibility was defined as: 1. Sea floor clearly visible (depth shallow) 2. Sea floor visible but obscured by turbidity (depth variable) 3. Sea floor not visible but clear water (depth likely > 5m) 4. Sea floor not visible and turbid water (depth variable)

Glare was a subjective estimate of the percentage area of the observers’ view affected (0%, <25%, 25-50% and >50%).

The distribution of the group size estimates for the dugong detections in the *aerial imagery* is given in *Figure 1*. Dugong group size was subsequently binned to account the heavy right skew on the distribution of group size estimates. The group size binning was 1, 2-4, and 5+ animals per group.

The distribution of water visibility values across the 96 dugong detections in the *aerial imagery*, by each of the three observers, is given in *Figure 2*. The distribution of glare values in the *aerial imagery* with dugong detections, by each of the observers, is given in *Figure 3*.

The distribution of group sizes across the 96 detections of dugongs, by each of the three observers, is given in *Figure 4*.


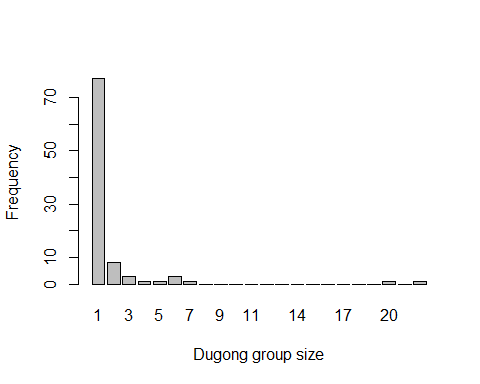


**Figure 1** Histogram of (unbinned) group size estimates across the 96 dugong group detections found within the aerial imagery data.


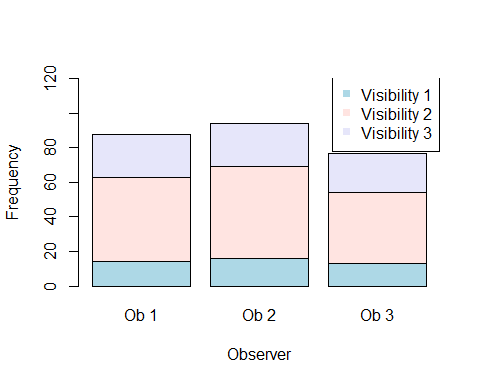


**Figure 2** Histogram of water visibility (turbidity) scores across the 96 dugong group detections made by each of the three observers (Ob 1, Ob 2 and Ob 3). Note, there was no water visibility type 4 observed in this study.


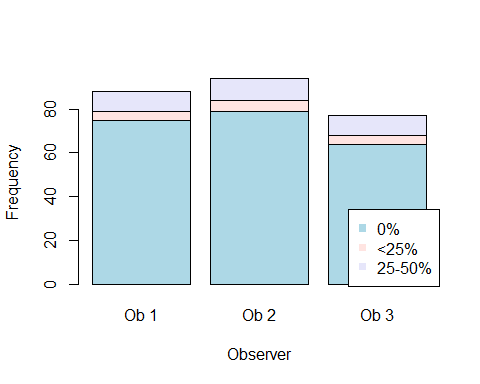


**Figure 3** Histogram of glare scores across the 96 dugong group detections across each of the three observers (Ob 1, Ob 2 and Ob 3). Note, there was no glare type ‘>50%’ observed in this set of aerial images.


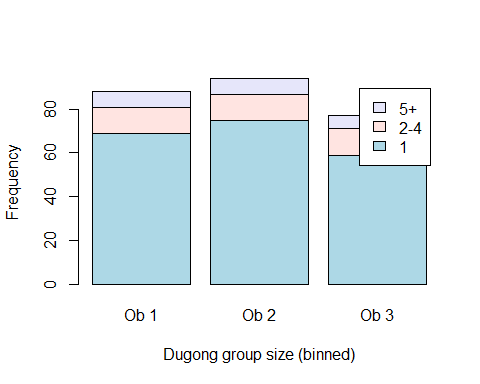


**Figure 4** Histogram of group size estimates across the 96 dugong group detections across each of the three observers (Ob 1, Ob 2 and Ob 3).

Model selection was undertaken using a small-sample Akaike’s Information Criterion (AIC_c_), which is an information-theoretic approach to model-selection, with a correction for small sample size (Burnham and Anderson 2002).

Model selection indicated that the best model to estimate the perception probability for detecting dugong groups was individual ID of the image reviewer/observer analyst (*Table 1*, model ‘4’), with no other covariates influencing perception probability.

**Table 1** Model selection for the Huggins models to estimate perception probability for the image survey based on the relative AICc, Akaike weights, and deviance calculated.

|  | p | c | model | npar | AICc | DeltaAICc | weight | Deviance |
| --- | --- | --- | --- | --- | --- | --- | --- | --- |
| 4 | ~observer**^1^** |  | p(~observer)c() | 3 | 176.05 | 0.00 | 0.4598 | 389.22 |
| 5 | ~Turbidity * observer |  | p(~Turbidity * observer)c() | 9 | 177.46 | 1.41 | 0.2273 | 378.07 |
| 8 | ~size.bin * observer |  | p(~size.bin * observer)c() | 9 | 177.95 | 1.90 | 0.1779 | 385.90 |
| 3 | ~size.bin * observer + Glare |  | p(~size.bin * observer + Glare)c() | 11 | 180.66 | 4.61 | 0.0460 | 384.30 |
| 2 | ~Glare * observer + Turbidity |  | p(~Glare * observer + Turbidity)c() | 11 | 181.09 | 5.03 | 0.0371 | 377.38 |
| 13 | ~Turbidity * observer + Glare |  | p(~Turbidity * observer + Glare)c() | 11 | 181.46 | 5.41 | 0.0308 | 377.76 |
| 7 | ~Glare * observer |  | p(~Glare * observer)c() | 9 | 184.35 | 8.30 | 0.0073 | 384.96 |
| 12 | ~size.bin |  | p(~size.bin)c() | 3 | 184.48 | 8.42 | 0.0068 | 404.99 |
| 10 | ~Turbidity |  | p(~Turbidity)c() | 3 | 185.88 | 9.83 | 0.0034 | 399.05 |
| 6 | ~Turbidity * observer + Glare * observer |  | p(~Turbidity * observer + Glare * observer)c() | 15 | 187.26 | 11.21 | 0.0017 | 374.75 |
| 9 | ~size.bin * observer + Turbidity * observer + Glare * observer |  | p(~size.bin * observer + Turbidity * observer + Glare * observer)c() | 21 | 187.75 | 11.70 | 0.0013 | 368.88 |
| 1 | ~1 |  | p(~1)c() | 1 | 189.94 | 13.89 | 0.0004 | 407.18 |
| 11 | ~Glare |  | p(~Glare)c() | 3 | 192.77 | 16.72 | 0.0001 | 405.94 |

**^1^** In traditional Huggins mark-recapture models, ‘observer’ refers to the sampling period, and is given as ‘time’ in Mark/rMark.

Perception probability estimates per observer, according to the best model (model 4 in *Table 1*) is given in *Table 2*.

**Table 2** Perception probability estimates for detecting dugongs, per three observers, for the aerial imagery data, according to the best model (model 4 in Table 1).

| observer | perc bias est | perc bias SE | perc bias CV |
| --- | --- | --- | --- |
| Ob 1 | 0.916 | 0.028 | 0.031 |
| Ob 2 | 0.979 | 0.015 | 0.015 |
| Ob 3 | 0.802 | 0.041 | 0.051 |
| combined | 1.000 | 0.000 | 0.000 |

### Human observers on the occupied survey flights

For the occupied flights, a team of four observers sat two on each side of the aircraft. The two observers on each side of the aircraft were visually and acoustically isolated from each other, and thus were assumed to be independent. The two front seat observers were considered to be the more experienced within the port or starboard observer pairs. There were 287 dugong detections in total by the four observers during the occupied survey.

In addition to testing front versus back observers (ostensibly, observer ‘experience’), the aircraft side (port and starboard) and the effect of dugong group size (binned 1, 2-4 and 5+) were also included in the double-observer analysis.

The distribution of the group size estimates for the dugong detections during the *occupied survey* is given in *Figure 5*. Dugong group size was subsequently binned to account the heavy right skew on the distribution of group size estimates. The group size binning was 1, 2-4, and 5+ animals per group.

The distribution of dugong detections between Port and Starboard, for the front and back observers, during the occupied flights is given in *Figure 6*. The distribution of binned group sizes across the 287 detections of dugongs, by the front and back observers, is given in *Figure 7*.


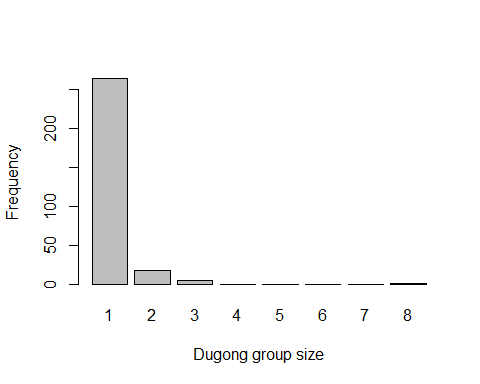


**Figure 5** Histogram of (unbinned) group size estimates across the 287 dugong group detections made during the occupied aerial survey.


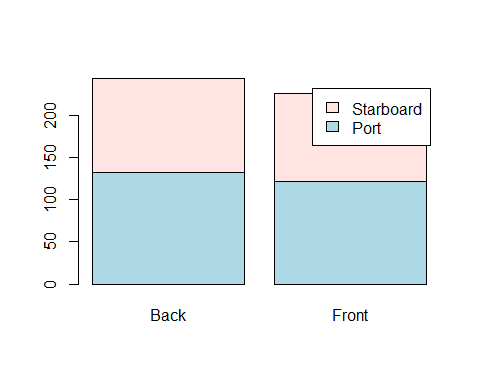


**Figure 6** Histogram of of detections across the ‘Back’ and ‘Front’ observers and the side of the aircraft (‘Port’ and ‘Starboard’).


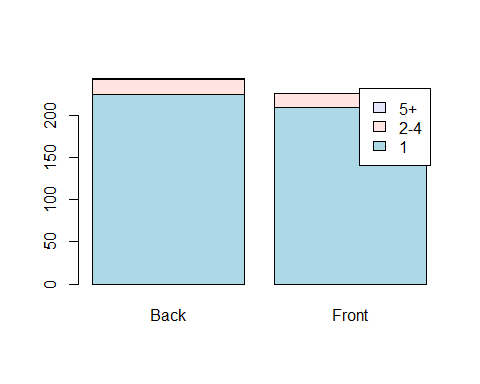


**Figure 7** Histogram of of detections across the ‘Back’ and ‘Front’ observers for each group size bin.

Model selection indicated that the best model to estimate the perception probability for detecting dugong groups was individual ID of the image reviewer/observer analyst (*Table 3*, model ‘2’), with no other covariates influencing perception probability.

**Table 3** Model selection for the Huggins models to estimate perception probability for the observers participating in the occupied aerial survey, based on the relative AICc, Akaike weights, and deviance calculated.

|  | p | c | model | npar | AICc | DeltaAICc | weight | Deviance |
| --- | --- | --- | --- | --- | --- | --- | --- | --- |
| 2 | ~observer |  | p(~observer)c() | 2 | 520.90 | 0.00 | 0.5394 | 2292.52 |
| 1 | ~1 |  | p(~1)c() | 1 | 522.02 | 1.12 | 0.3085 | 2295.66 |
| 3 | ~observer * side |  | p(~observer * side)c() | 4 | 524.89 | 3.98 | 0.0736 | 2292.46 |
| 4 | ~size.bin * observer |  | p(~size.bin * observer)c() | 6 | 525.01 | 4.11 | 0.0692 | 2139.69 |
| 5 | ~size.bin * observer + observer * side |  | p(~size.bin * observer + observer * side)c() | 8 | 529.01 | 8.11 | 0.0094 | 2139.59 |

Perception probability estimates per observer, according to the best model (model 2 in *Table 3*) is given in *Table 4*.

**Table 4** Perception probability estimates for detecting dugongs, per front and back observer, on the occupied flights, according to the best model (model 2 in Table 3.

| observers | Perception prob | Perception prob SE | Perception prob CV |
| --- | --- | --- | --- |
| front | 0.750 | 0.028 | 0.037 |
| back | 0.810 | 0.026 | 0.032 |
| combined | 0.952 | 0.009 | 0.010 |

## References

Burnham, K. P., and D. R. Anderson. 2002. *Model Selection and Multimodel Inference: A Practical Information-Theoretic Approach.* Book. Second Edition. Springer-Verlag New York. <https://doi.org/10.1007/b97636>.

Huggins, R. M. 1989. “On the Statistical Analysis of Capture Experiments.” Journal Article. *Biometrika* 76 (1): 133–40. <https://doi.org/10.1093/biomet/76.1.133>.

———. 1991. “Some Practical Aspects of a Conditional Likelihood Approach to Capture Experiments.” Journal Article. *Biometrics* 47 (2): 725–32. <https://doi.org/10.2307/2532158>.

Laake, J. L. 2013. “RMark: An r Interface for Analysis of Capture-Recapture Data with MARK.” {AFSC} Processed Rep. 2013-01. Seattle, WA: Alaska Fish. Sci. Cent., NOAA, Natl. Mar. Fish. Serv. <http://www.afsc.noaa.gov/Publications/ProcRpt/PR2013-01.pdf>.

Marsh, H., and D. F. Sinclair. 1989. “Correcting for Visibility Bias in Strip Transect Aerial Surveys of Aquatic Fauna.” Journal Article. *Journal of Wildlife Management* 53: 1017–24.

Nichols, James D., James E. Hines, John R. Sauer, Frederick W. Fallon, Jane E. Fallon, and Patricia J. Heglund. 2000. “A Double-Observer Approach for Estimating Detection Probability and Abundance from Point Counts.” Journal Article. *The Auk* 117 (2): 393–408. <https://doi.org/10.1093/auk/117.2.393>.

Otis, David L., Kenneth P. Burnham, Gary C. White, and David R. Anderson. 1978. “Statistical Inference from Capture Data on Closed Animal Populations.” Journal Article. *Wildlife Monographs*, no. 62: 3–135. <http://pubs.er.usgs.gov/publication/70119899>.

R Core Team. 2020. *R: A Language and Environment for Statistical Computing*. Vienna, Austria: R Foundation for Statistical Computing. <https://www.R-project.org/>.

White, Gary C., and Kenneth P. Burnham. 1999. “Program MARK: Survival Estimation from Populations of Marked Animals.” Journal Article. *Bird Study* 46 (sup1): S120–39. <https://doi.org/10.1080/00063659909477239>.
