# Supplementary material for: Drone images afford more detections of marine wildlife than real-time observers during simultaneous large-scale surveys
Source: PeerJ. 2023 Nov 3;11:e16186. doi: 10.7717/peerj.16186 (PMC10629383; doi:10.7717/peerj.16186)
Supplement: Supplemental Information 4 [file peerj-11-16186-s004.docx]

**Supplemental Data S4**

# Generalised linear mixed models: Output

Note: All models had an offset term to account for the area surveys and transect and flight number were crossed random effects.

Family: Tweedie

Link function: log

Formula: **DugCount ~ WaterVis + Sea_State + Platform**

Parametric coefficients:

|  | Estimate | Std. Error | t value | Pr(>\|t\|) |  |
| --- | --- | --- | --- | --- | --- |
| (Intercept) | -0.515 | 0.3711 | -1.388 | 0.1656 |  |
| WaterVis2 | 0.4314 | 0.2273 | 1.898 | 0.05806 | . |
| WaterVis3 | -0.7495 | 0.271 | -2.766 | 0.00582 | ** |
| Sea_State | -0.2305 | 0.1138 | -2.027 | 0.04306 | * |
| PlatformU | 0.295 | 0.1601 | 1.842 | 0.06582 | . |

Signif. codes: 0 ‘***’ 0.001 ‘**’ 0.01 ‘*’ 0.05 ‘.’ 0.1 ‘ ’ 1

Family: Tweedie

Link function: log

Formula: **NoGroup ~ BotVis**

Parametric coefficients:

|  | Estimate | Std. Error | t value | Pr(>\|t\|) |  |
| --- | --- | --- | --- | --- | --- |
| (Intercept) | -1.3574 | 0.2678 | -5.069 | 5.04E-07 | *** |
| WaterVis2 | 0.2902 | 0.2002 | 1.45 | 0.14753 |  |
| WaterVis3 | -0.6089 | 0.2252 | -2.704 | 0.00701 | ** |

Signif. codes: 0 ‘***’ 0.001 ‘**’ 0.01 ‘*’ 0.05 ‘.’ 0.1 ‘ ’ 1

Family: truncated_poisson

Link function: log

Formula: **Group_size ~ Sea_State + BotVis + Cloud_Cover * Platform**

Conditional model:

|  | Estimate | Std. Error | z value | Pr(>\|z\|) |  |
| --- | --- | --- | --- | --- | --- |
| (Intercept) | -1.1062 | 0.4825 | -2.293 | 0.02186 | * |
| Sea_State | -0.2959 | 0.1353 | -2.187 | 0.02871 | * |
| WaterVis2 | 0.5738 | 0.217 | 2.644 | 0.0082 | ** |
| WaterVis3 | -0.4592 | 0.318 | -1.444 | 0.14874 |  |
| Cloud_Cover | -1.2358 | 0.4437 | -2.785 | 0.00535 | ** |
| PlatformU | 0.8084 | 0.1517 | 5.33 | 9.84E-08 | *** |
| Cloud_Cover:PlatformU | 1.0825 | 0.4525 | 2.392 | 0.01675 | * |

Signif. codes: 0 ‘***’ 0.001 ‘**’ 0.01 ‘*’ 0.05 ‘.’ 0.1 ‘ ’ 1
